# P4HA1, a Prognostic Biomarker that Correlates With Immune Infiltrates in Lung Adenocarcinoma and Pan-Cancer

**DOI:** 10.3389/fcell.2021.754580

**Published:** 2021-12-13

**Authors:** Qi Zhao, Junfeng Liu

**Affiliations:** Department of Thoracic Surgery, The Fourth Hospital of Hebei Medical University, Shijiazhuang, China

**Keywords:** TCGA, pan-cancer, prognostic biomarker, macrophages, immunosuppressive microenvironment

## Abstract

**Objective:** Prolyl 4-hydroxylase, alpha polypeptide I (P4HA1), a key enzyme in collagen synthesis, comprises two identical alpha subunits and two beta subunits. However, the immunomodulatory role of P4HA1 in tumor immune microenvironment (TIME) remains unclear. This study aimed to evaluate the prognostic value of P4HA1 in pan-cancer and explore the relationship between P4HA1 expression and TIME.

**Methods:** P4HA1 expression, clinical features, mutations, DNA methylation, copy number alteration, and prognostic value in pan-cancer were investigated using the Cancer Genome Atlas (TCGA) and Genotype-Tissue Expression data. Pathway enrichment analysis of P4HA1 was performed using R package “clusterProfiler.” The correlation between immune cell infiltration level and P4HA1 expression was analyzed using three sources of immune cell infiltration data, including ImmuCellAI database, TIMER2 database, and a published work.

**Results:** P4HA1 was substantially overexpressed in most cancer types. P4HA1 overexpression was associated with poor survival in patients. Additionally, we discovered that P4HA1 expression was positively associated with infiltration levels of immunosuppressive cells, such as tumor-associated macrophages, cancer-associated fibroblasts, nTregs, and iTregs, and negatively correlated with CD8^+^ T and NK cells in pan-cancer.

**Conclusions:** Our results highlighted that P4HA1 might serve as a potential prognostic biomarker in pan-cancer. P4HA1 overexpression is indicative of an immunosuppressive microenvironment. P4HA1 may be a potential target of immunotherapy.

## Introduction

Cancer is the main cause of human death (Siegel et al., 2020). Although huge progress has been made in cancer-related treatment technologies in recent years, patient prognosis remains very low, mainly because most patients are diagnosed in the final stage and lack effective specific therapeutic targets. Onco-immunotherapies, such as immune checkpoint blockade inhibitors, have revolutionized the prognosis of cancer patients, but most patients remain insensitive to immunotherapy (Mainini et al., 2021). Therefore, it is urgent to investigate the underlying mechanism of tumors and determine novel key biomarkers and potential immunotherapy targets for tumor patients.

Recent studies have demonstrated that immunosuppressive tumor immune microenvironment (TIME) is critical in developing tumor progression and weakening the response of tumor patients to immunotherapy (Taki et al., 2021; Wang et al., 2021). Immune cells in TIME were remodeled and lost their original function. For instance, tumor-associated macrophages (TAMs), particularly M2-like TAMs, were remolded directly or indirectly by tumor cells and subsequently played an immunosuppressive and tumor-promoting role (Choi et al., 2018; Deepak et al., 2020; Mangogna et al., 2021; Zhou et al., 2021). However, the number of biomarker genes which can predict the status of TIME is small.

Prolyl 4-hydroxylase, alpha polypeptide I (P4HA1), a key enzyme in collagen synthesis, comprises two identical alpha subunits and two beta subunits. Collagen is the major component of tumor microenvironment and participates in cancer fibrosis, which increases tumor tissue stiffness, regulates tumor immunity, and promotes metastasis (Discher et al., 2017; Yamauchi et al., 2018). P4HA1 was reported to play an unfavorable prognostic marker in glioma and promote migration and invasion of glioma by accelerating EMT process (Zhu et al., 2021). Another study revealed that P4HA1 hypoxia-induced P4HA1 overexpression could promote colorectal cancer progression (Zhang et al., 2021). However, the immunomodulatory role of P4HA1 in pan-cancer is insufficient.

This study comprehensively analyzed the role of P4HA1 using multi-omics data from TCGA database for 33 cancers, including expression, clinical features, prognostic values, DNA methylation, copy number alteration (CNA), and mutation status of P4HA1. Further investigation was conducted on the relationship between P4HA1 expression and TIME. P4HA1 overexpression predicted immunosuppressive TIME, which may cause poorer survival in tumor patients with P4HA1 overexpression. As a result, targeting P4HA1 may be potential cancer immunotherapy.

## Materials and Methods

### Data Collection

The expression profiles and clinical information of the Cancer Genome Atlas (TCGA), Genotype-Tissue Expression (GTEx), and Cancer Cell Line Encyclopedia (CCLE) were downloaded from UCSC Xena (https://xenabrowser.net/datapages/) database. The immune cell infiltration data of TCGA were downloaded from a published study (Thorsson et al., 2018), TIMER2 database (http://timer.comp-genomics.org/) (Li et al., 2020a), and ImmuCellAI database (http://bioinfo.life.hust.edu.cn/ImmuCellAI#!/) (Miao et al., 2020). The methylation and CNA of P4HA1 were downloaded from cBioPortal database (https://www.cbioportal.org/) (Cerami et al., 2012).

### Prognostic Analysis

Kaplan-Meier analyses and univariate Cox regression (UniCox) were conducted to explore P4HA1 impact on the survival of patients considering pan-cancer analysis using R package “survminer” and “survival.” Overall survival (OS) and disease-specific survival (DSS) were evaluated.

### Correlation and Gene Set Enrichment Analysis

The correlation analysis between P4HA1 expression level and all protein-coding genes was conducted using TCGA pan-cancer data, and Pearson’s correlation coefficient was further calculated. The genes correlated with P4HA1 (*p* < 0.05) were ranked and subjected to GSEA analysis. The analysis was performed using R package “clusterProfiler.”.

### Drug Response Analysis

IC50 values of 192 drugs and gene expression profiles for 809 cell lines were downloaded from Genomics of Drug Sensitivity in Cancer database (GDSC: https://www.cancerrxgene.org/). The correlation between P4HA1 expression and IC50 values of 192 drugs was analyzed.

## Results

### Expression of P4HA1 in Pan-Cancer

**FIGURE 1:**
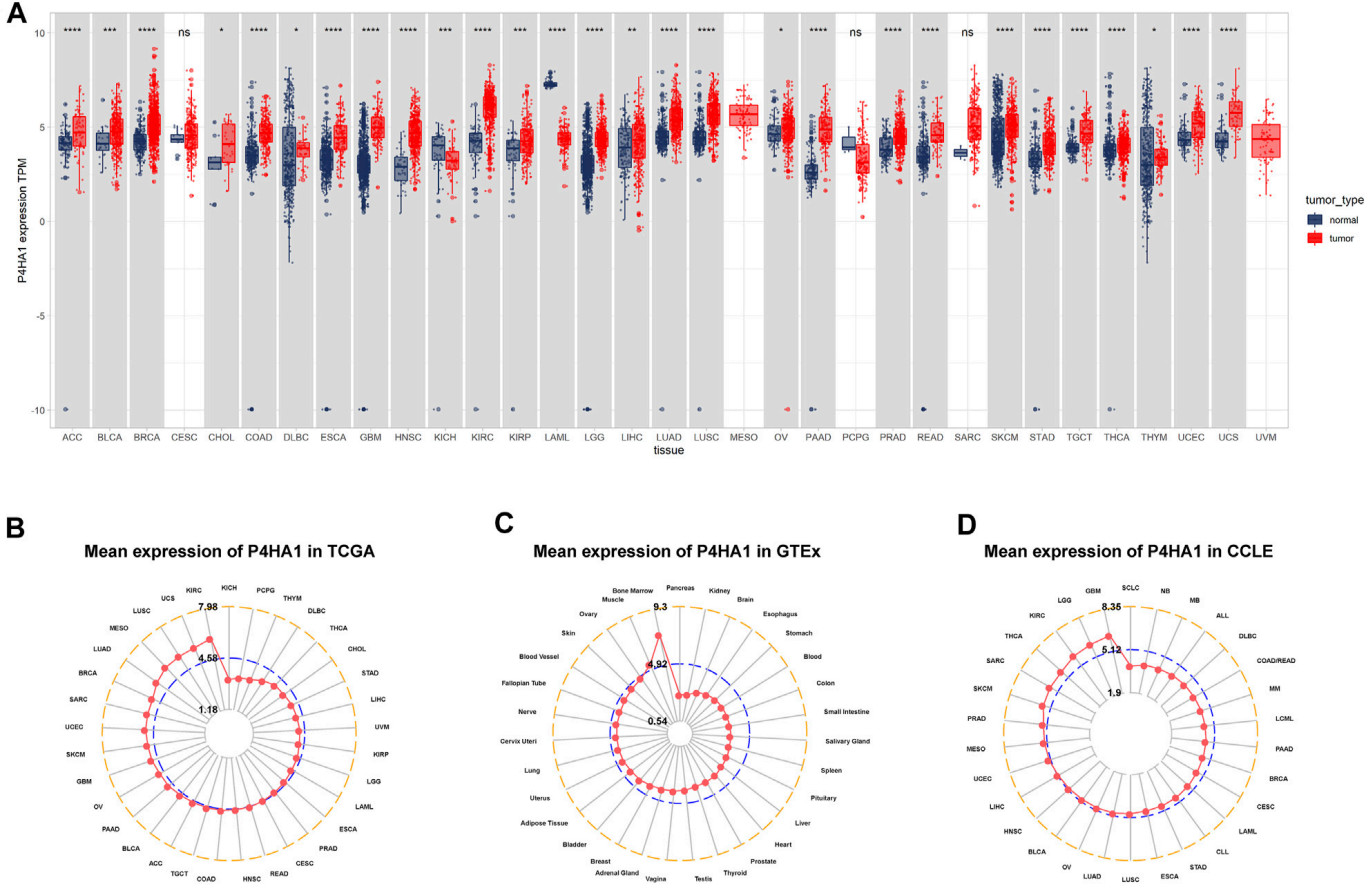
Expression of P4HA1 in pan-cancer. **(A)** pan-cancer expression of P4HA1. **(B)** P4HA1 expression in tumor tissues from TCGA cohort. **(C)** P4HA1 expression in normal tissues from GTEx cohort. **(D)** P4HA1 expression in cancer cell lines from CCLE cohort. *p < 0.05, **p < 0.01, ***p < 0.001, ****p < 0.0001.

We first assessed the expression of P4HA1 using TCGA, GTEx, and CCLE data. We found that P4HA1 was overexpressed in 26 of 33 cancers types, including ACC, BLCA, BRCA, CHOL, COAD, DLBC, ESCA, GBM, HNSC, KIRC, KIRP, LGG, LIHC, LUAD, LUSC, OV, PAAD, PRAD, READ, SKCM, STAD, TGCT, THCA, THYM, UCEC, and UCS. In addition, P4HA1 expression was low in only two tumor types, such as KICH and LAML (Figure 1A). To compare P4HA1 expression only in tumor tissues, we found that P4HA1 was highly expressed in KIRC and the lowest in KICH (Figure 1B). In normal tissues from GTEx database, the results revealed that P4HA1 expression was the highest in bone marrow tissues and the lowest in pancreas (Figure 1C). As for tumor cell lines, we proved that P4HA1 expression was the highest in GBM cell lines using data from CCLE database (Figure 1D).

**FIGURE 2:**
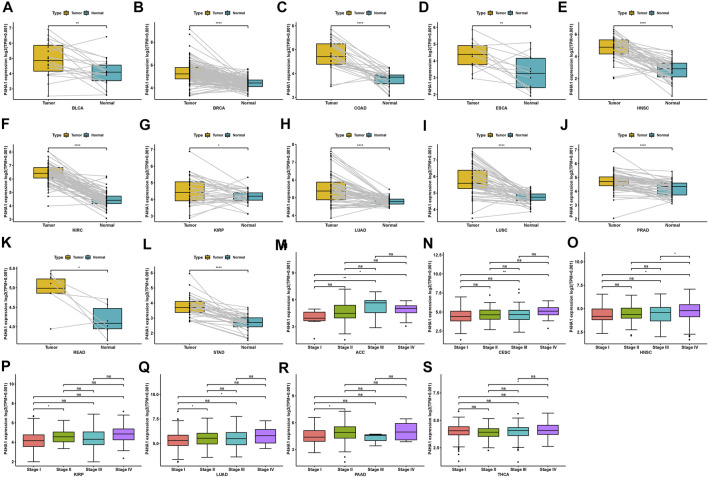
Expression of P4HA1 in paired tumor and adjacent normal tissues and various tumor stages. **(A–L)** P4HA1 expression in paired tumor and adjacent normal tissues from TCGA in indicated tumor types. **(M–S)** P4HA1 expression in various tumor stages in indicated tumor types. *p < 0.05, **p < 0.01, ***p < 0.001, ****p < 0.0001.

Regarding tumor and adjacent normal tissues in TCGA cohort, P4HA1 was also overexpressed in 12 cancers, such as BLCA, BRCA, COAD, ESCA, HNSC, KIRC, KIRP, LUAD, LUSC, PRAD, READ, and STAD (Figures 2A–L). Additionally, we investigated P4HA1 expression at various tumor stages. The results demonstrated that P4HA1 expression was elevated in the relatively worse tumor stages in ACC, CESC, HNSC, KIRP, LUAD, PAAD, and THCA (Figures 2M–S).

### Gene Alteration of P4HA1 in Pan-Cancer

**FIGURE 3:**
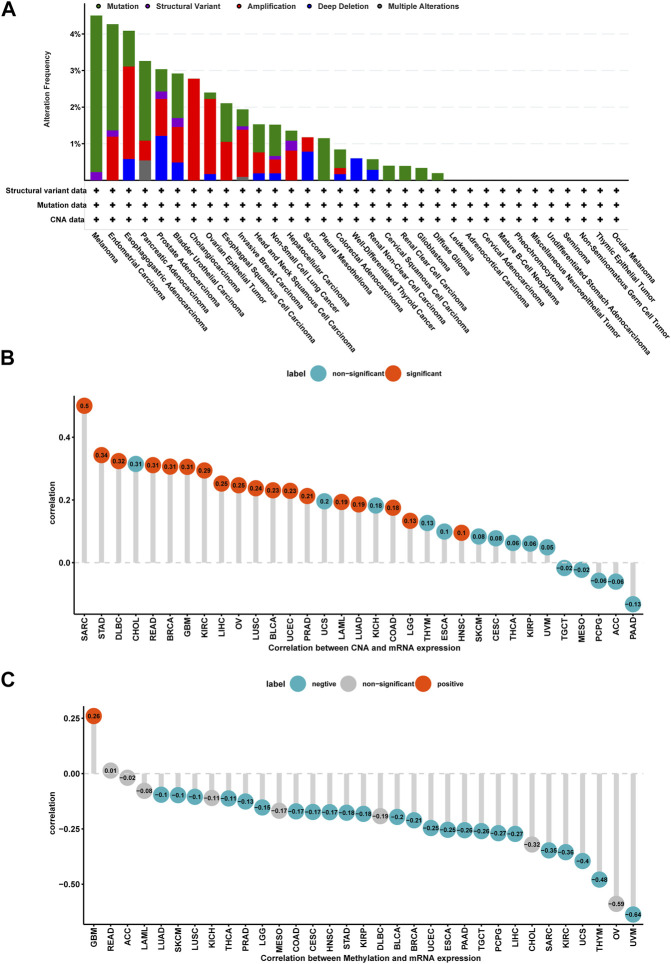
Gene alteration of P4HA1. **(A)** The mutation and CNA status of P4HA1 in TCGA-pan-cancer. **(B)** The correlation between P4HA1 expression and CNA. **(C)** The correlation between P4HA1 expression and DNA methylation.

Mutation, DNA methylation, and CNA are factors that influence gene expression; therefore, we assessed them for P4HA1. We found that the highest alteration frequency of P4HA1 (>4%) was observed in melanoma patients, in which “Mutation” was the primary type (Figure 3A). For the correlation between P4HA1 and CNA, we discovered that P4HA1 expression was positively correlated with CNA in 18 of 33 tumor types (Figure 3B), indicating that elevated CNA was one of the main causes of high P4HA1 expression in pan-cancer. In addition, we found that the promoter methylation level of P4HA1 was negatively linked to P4HA1 expression in 24 of 33 tumor types, which also induced high P4HA1 expression (Figure 3C).

### Prognostic Value of P4HA1

**FIGURE 4:**
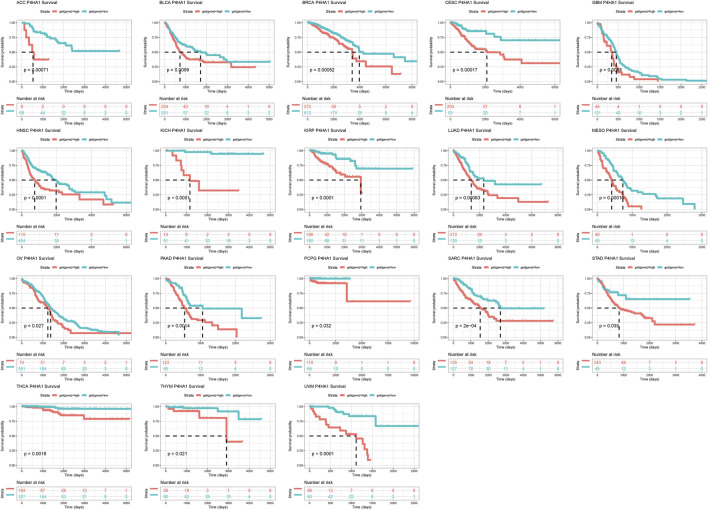
Prognostic value of P4HA1. Kaplan-Meier OS analysis of P4HA1 in TCGA pan-cancer in indicated tumor types.

To evaluate the prognostic value of P4HA1 in predicting the prognosis of patients with tumors, univariate Cox regression (UniCox) and Kaplan-Meier survival analyses were conducted. Kaplan-Meier OS analysis proved that an elevated P4HA1 expression predicted worse OS of patients with ACC, BLCA, BRCA, CESC, GBM, HNSC, KICH, KIRP, LUAD, MESO, OV, PAAD, PCPG, SARC, STAD, YHCA, THYM, and UVM (Figure 4).

**FIGURE 5:**
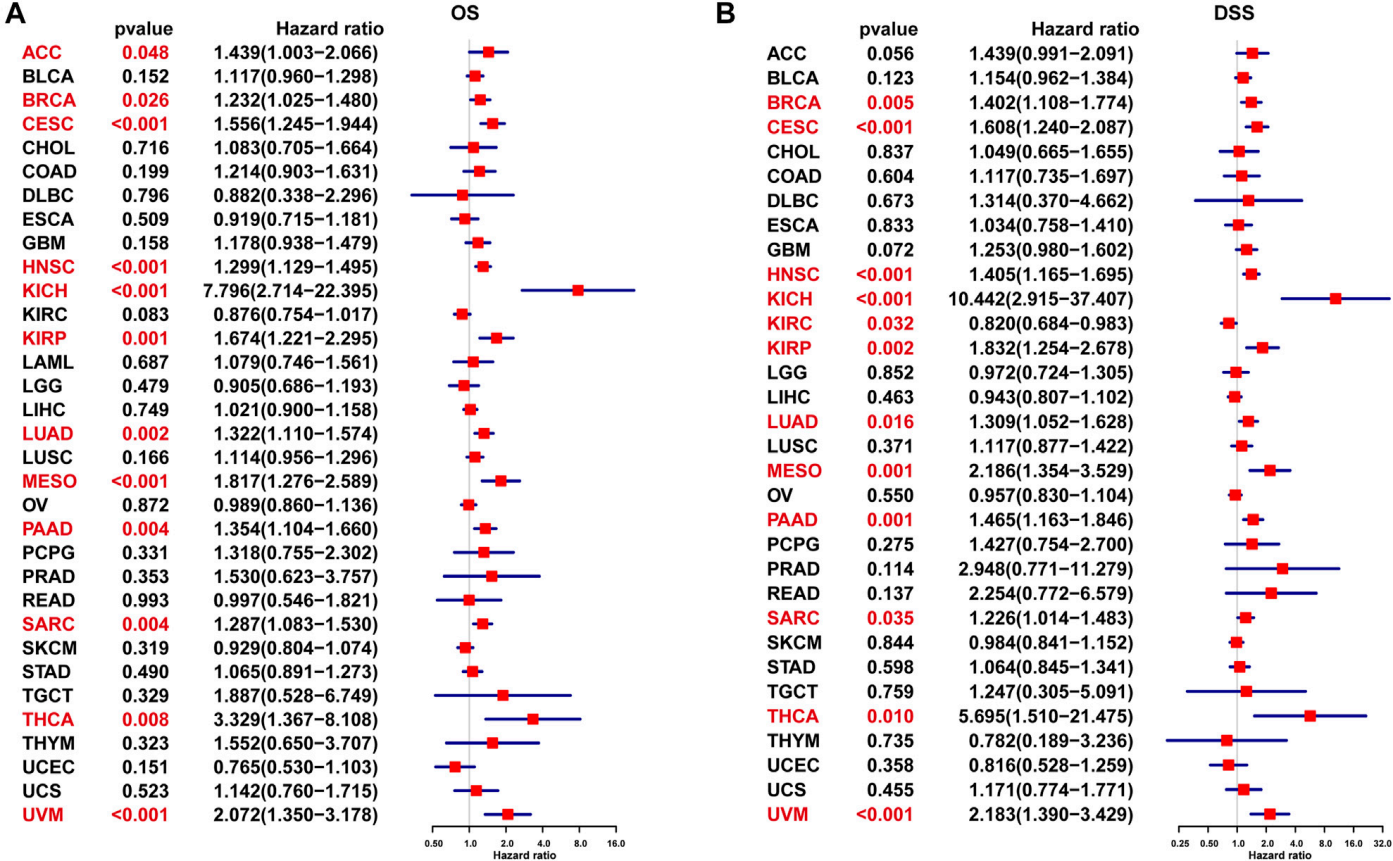
UniCox analysis of P4HA1. **(A)** The UniCox OS analysis of P4HA1 in TCGA pan-cancer. **(B)** The UniCox DSS analysis of P4HA1 in TCGA pan-cancer. Red color represents significant results (*p* < 0.05).

UniCox results indicated that P4HA1 was a risk factor for OS of patients with ACC, BRCA, CESC, HNSC, KICH, KIRP, LUAD, MESO, PAAD, SARC, THCA, and UVM (Figure 5A). Additionally, we assessed the prognostic value of P4HA1 in predicting DSS. The results indicated that a high P4HA1 expression predicted a worse DSS status in patients with BRCA, CESC, HNSC, KIRP, LUAD, MESO, PAAD, SARC, THCA, and UVM, but predicted a better DSS status in KIRC patients (Figure 5B).

### GSEA of P4HA1

**FIGURE 6:**
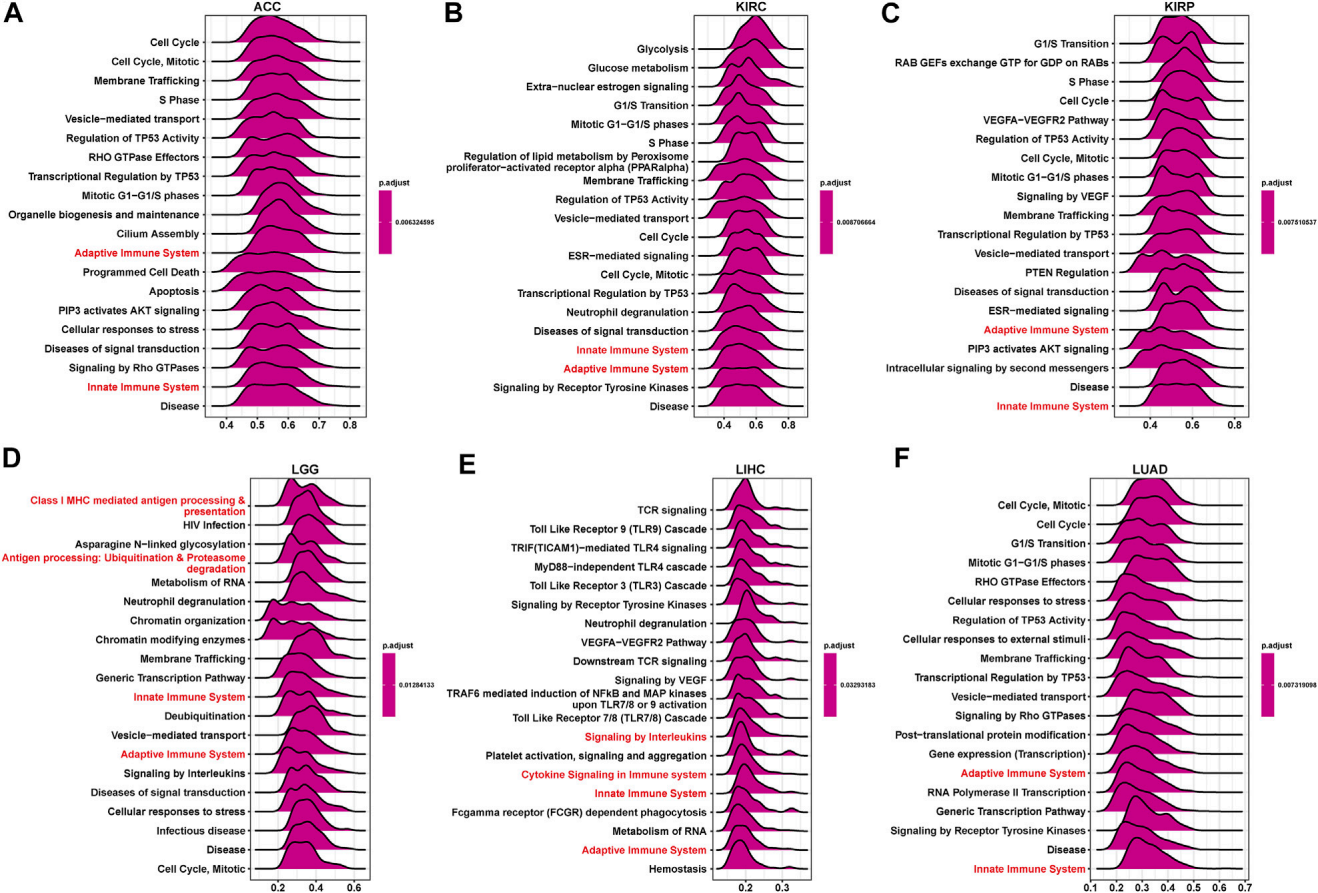
GSEA of P4HA1 in pan-cancer. **(A–F)** The top GSEA results in indicated tumor types (NES ≥1.5, adjusted *p*-value < 0.05). Red indicates immune regulation-related pathways.

**FIGURE 7:**
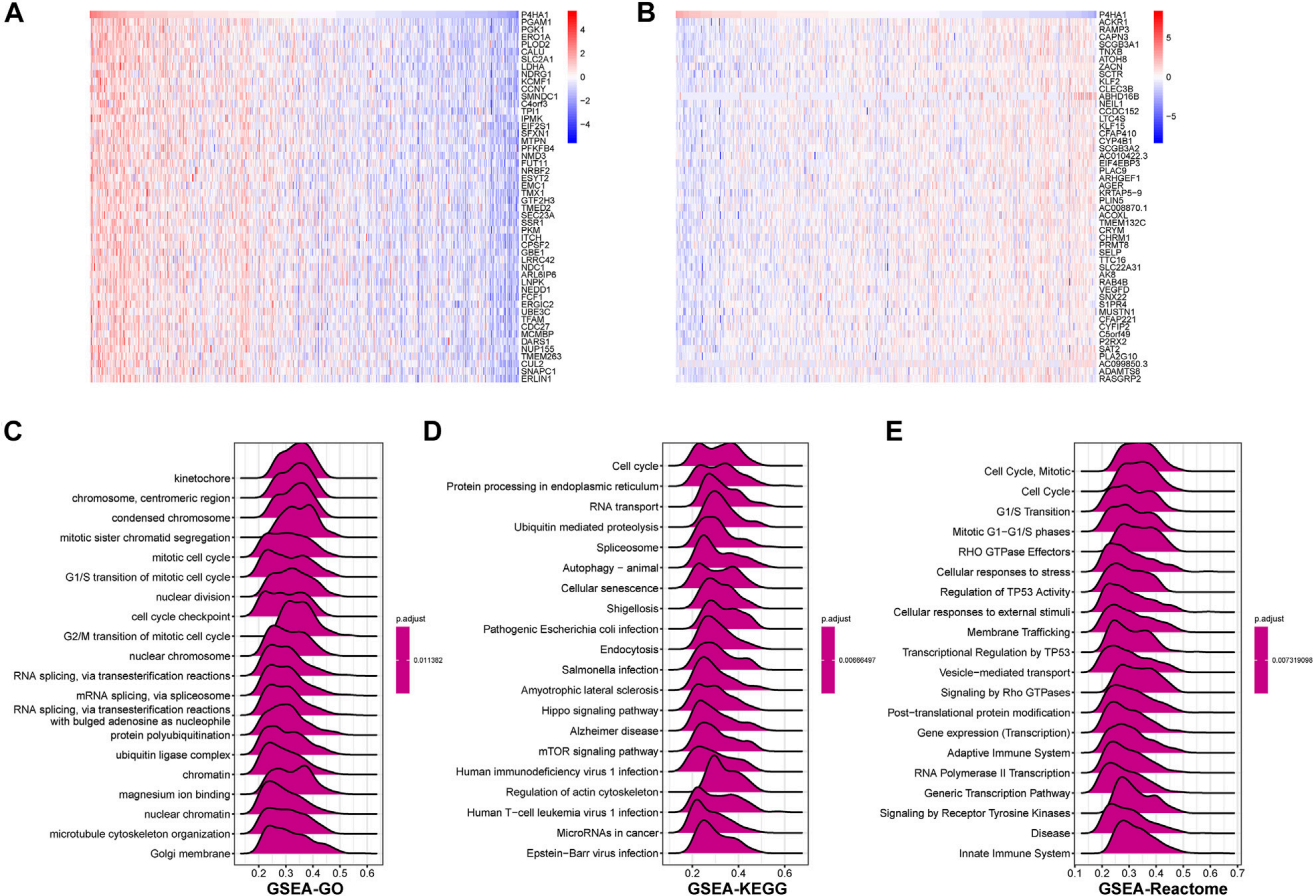
GSEA of P4HA1 in LUAD **(A)** The heatmap of top 50 genes positively correlated with P4HA1 expression. **(B)** The heatmap of top 50 genes negatively correlated with P4HA1 expression. **(C–E)** The top 20 GSEA results based on GO **(C)**, KEGG **(D)**, and Reactome **(E)** database shown in indicated tumor types.

To explore the possible pathway of P4HA1 involved in pan-cancer, we conducted GSEA based on Reactome pathway database. As displayed in Figure 6, GSEA results displayed that P4HA1 participated in immune regulation-related pathways in pan-cancer such as cytokine signaling in immune system, innate immune system, and adaptive immune system. In addition, to investigate potential pathways for participation of P4HA1 in LUAD, we conducted GSEA of P4HA1 using TCGA LUAD data. The correlation between all protein-coding genes and P4HA1 was calculated, and significant genes (*p* < 0.05) were selected to perform GSEA. The top 50 genes most positively and negatively correlated with P4HA1 were displayed in Figures 7A,B. For GSEA-GO results, we discovered that P4HA1 was enriched in most cell cycle-related terms (Figure 7C). For GSEA results based on KEGG and Reactome, P4HA1 was enriched in cell cycle, autophagy-animal, hippo signaling pathway, mTOR signaling pathway, adaptive immune system, and innate immune system pathways (Figures 7D,E). These results indicated that P4HA1-regulated cell cycle and immune-related pathways might contribute to poor survival of patients with tumors.

### Relationship Between P4HA1 Expression and Immune Cell Infiltration

**FIGURE 8:**
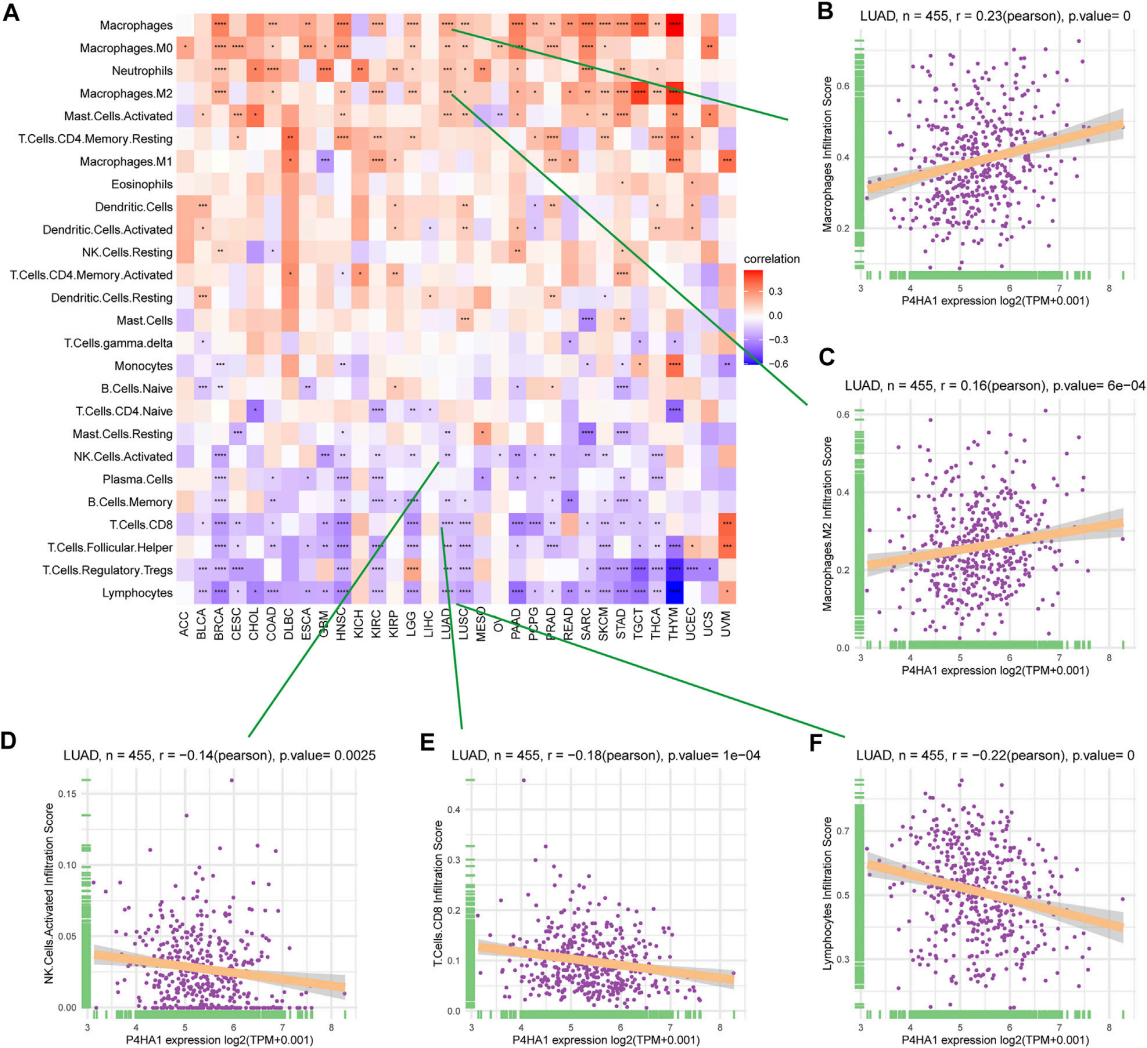
Immune infiltration analysis based on data from published research. **(A)** The relationship between P4HA1 expression and infiltration levels of 26 immune cells in pan-cancer. **(B)** The correlation between P4HA1 expression and infiltration levels of TAMs. **(C)** The correlation between P4HA1 expression and infiltration levels of M2-like TAMs. **(D)** The correlation between P4HA1 expression and infiltration levels of NK cells. **(E)** The correlation between P4HA1 expression and infiltration levels of CD8 T cells. **(F)** The correlation between P4HA1 expression and infiltration levels of lymphocytes. *p < 0.05, **p < 0.01, ***p < 0.001, ****p < 0.0001.

**FIGURE 9:**
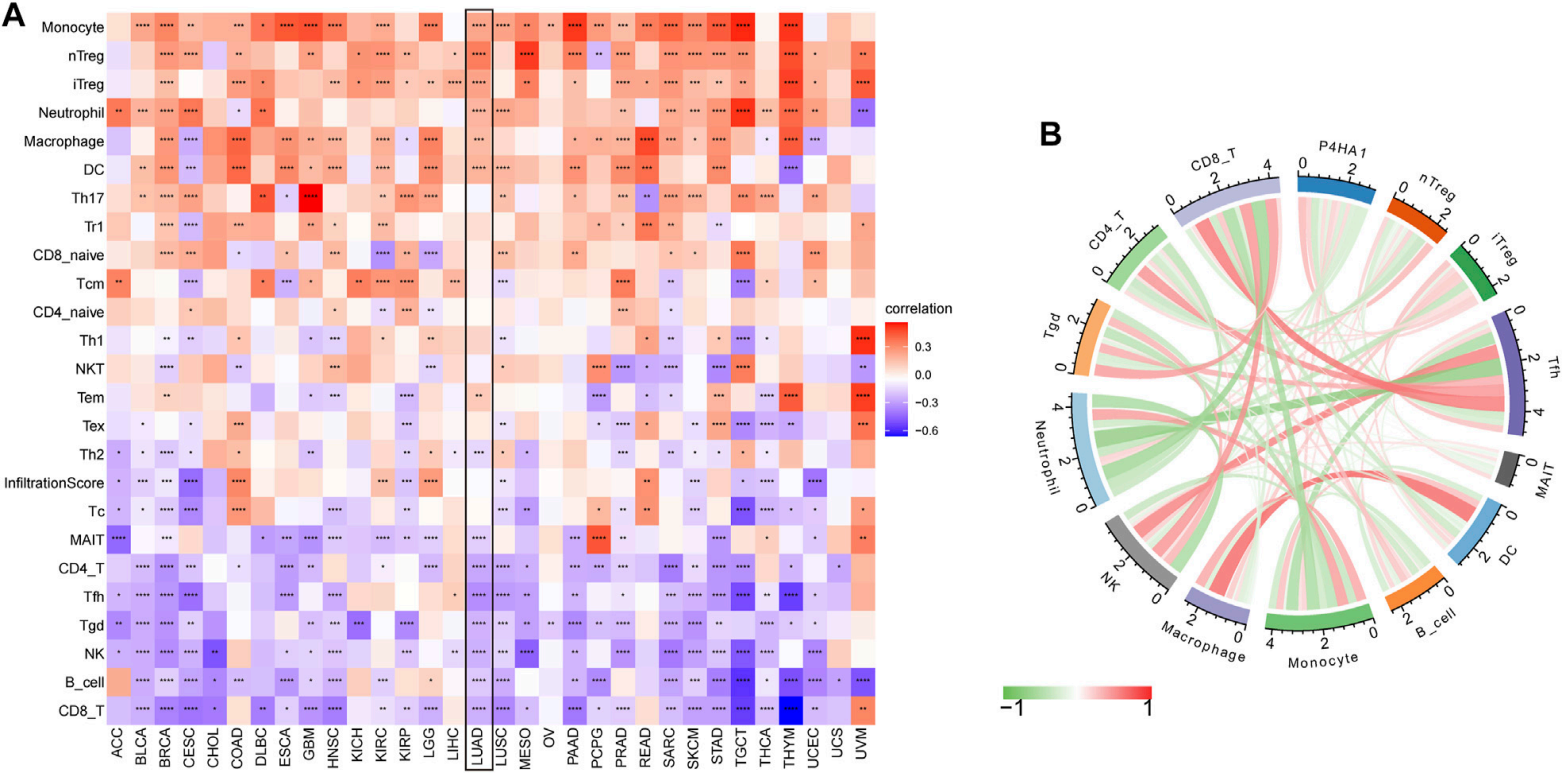
Immune infiltration analysis based on data from ImmuCellAI database. **(A)** The relationship between P4HA1 expression and infiltration levels of 24 immune cells in pan-cancer. **(B)** The correlation between P4HA1 expression and immune cells infiltration in LUAD. Red lines represent positive correlation, and green lines represent negative correlation, and the darker the color, the stronger the correlation. *p < 0.05, **p < 0.01, ***p < 0.001, ****p < 0.0001.

**FIGURE 10:**
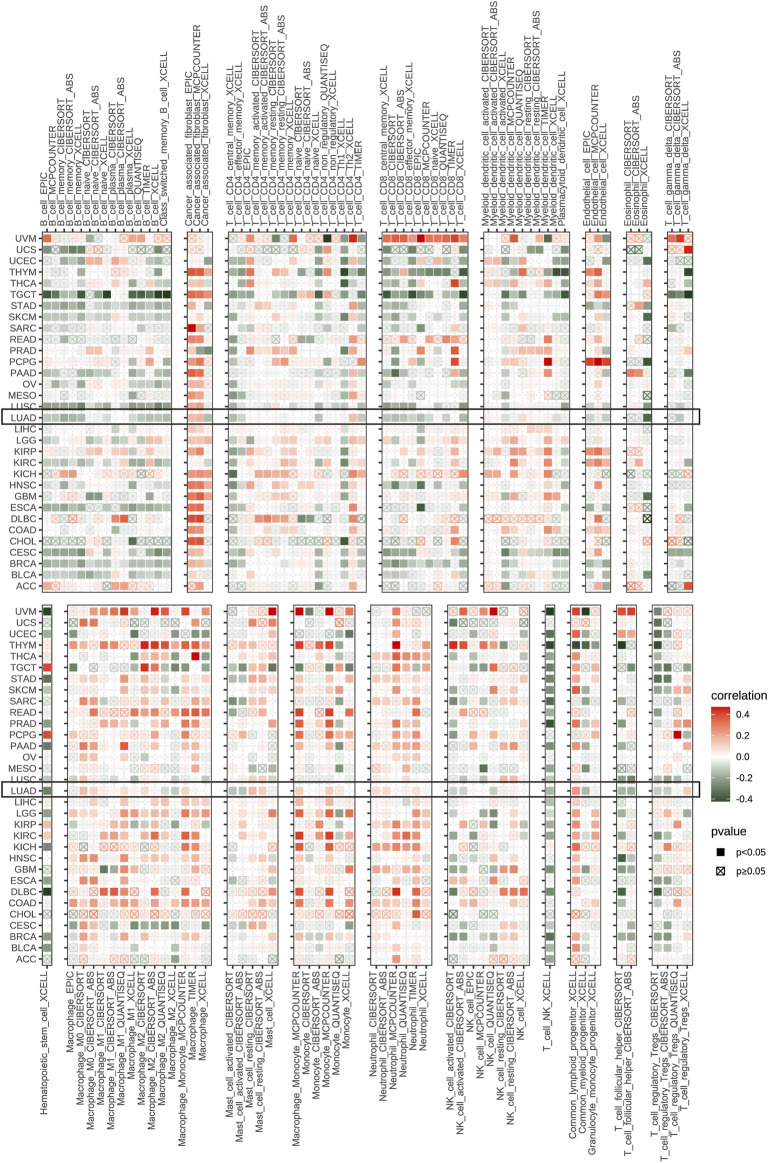
Immune infiltration analysis based on data from TIMER2 database. The relationship between P4HA1 expression and infiltration levels of immune cells in pan-cancer. Red represents positive correlation, green represents negative correlation, and the darker the color, the stronger the correlation. *p < 0.05, **p < 0.01, ***p < 0.001, ****p < 0.0001.

By analyzing the correlation between P4HA1 expression and immune cell infiltration using immune cell infiltration data from a published study (Thorsson et al., 2018), we found that P4HA1 was positively associated with TAMs infiltration in most tumor types, including LUAD (Figures 8A–C), but negatively associated with immune killer cells, such as activated NK cells, CD8 T cells, and lymphocytes in LUAD (Figures 8D–F). We also discovered a similar phenomenon using data from ImmuCellAI database. Immunosuppressive cells, like nTreg, iTreg, and TAMs, were positively linked to P4HA1 expression in pan-cancer (Figure 9A) and LUAD (Figure 9B). In contrast, immune killer cells, such as activated NK cells and CD8 T cells, were negatively associated with P4HA1 expression. Similar conclusions were also observed in the analysis results of data from TIMER2 database (Figure 10).

**FIGURE 11:**
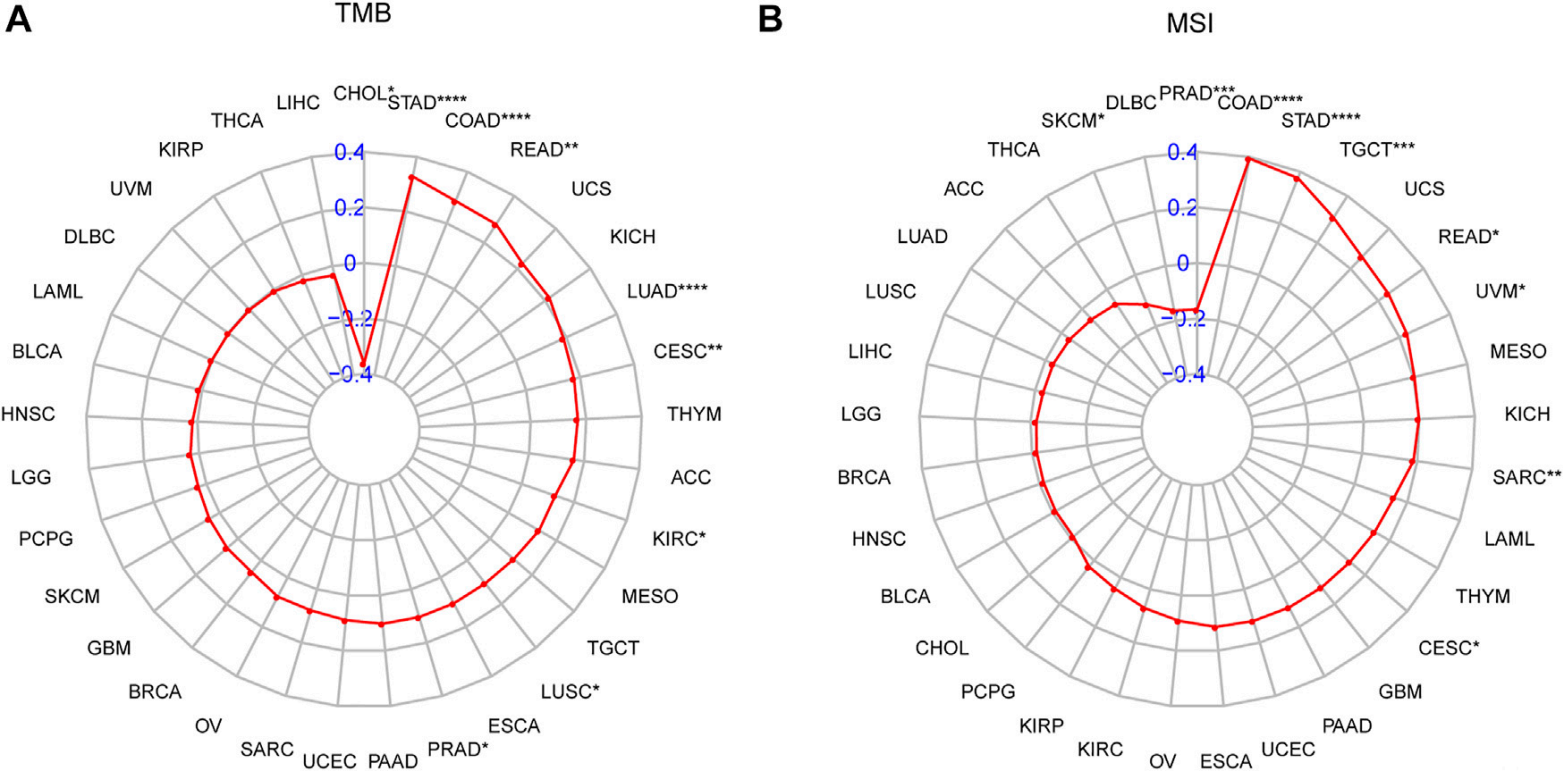
The correlation of P4HA1 expression with TMB and MSI. **(A–B)** Radar plots represent the correlation of P4HA1 expression with TMB **(A)** and MSI **(B)** in pan-cancer. The position of the red dot shows the correlation coefficient. *p < 0.05, **p < 0.01, ***p < 0.001, ****p < 0.0001.

**FIGURE 12:**
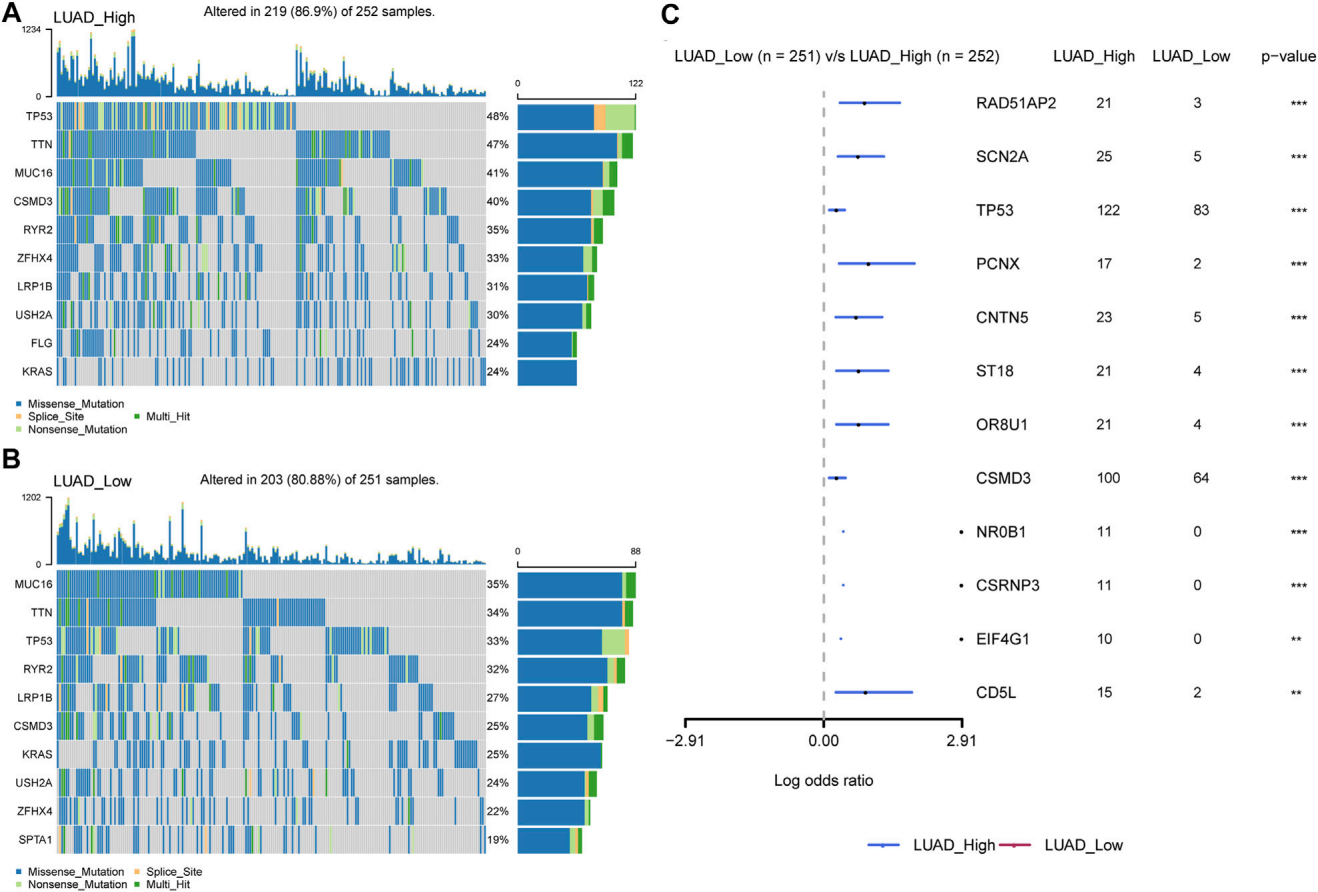
Gene mutation between high and low-P4HA1 groups in LUAD. **(A)** The top 10 genes with the highest mutation frequency in high-P4HA1 groups calculated by R package “maftools”. **(B)** The top 10 genes with the highest mutation frequency in low-P4HA1 groups. **(C)** The gene mutation differences between high and low-P4HA1 groups in LUAD.

In addition, we discovered that P4HA1 expression was positively linked to tumor mutation burden (TMB) and microsatellite instability (MSI) in several tumor types, demonstrating a potential association with immunotherapy efficacy (Figures 11A,B). Additionally, we divided LUAD patients into two groups according to their median expression of P4HA1 and compared the differences of gene mutations between the two groups. The results indicated that patients with high P4HA1 expression possess higher gene mutation frequency (high vs. low: 86.9 vs. 80.88%) than those with low P4HA1 expression in LUAD (Figures 12A,B).

**FIGURE 13:**
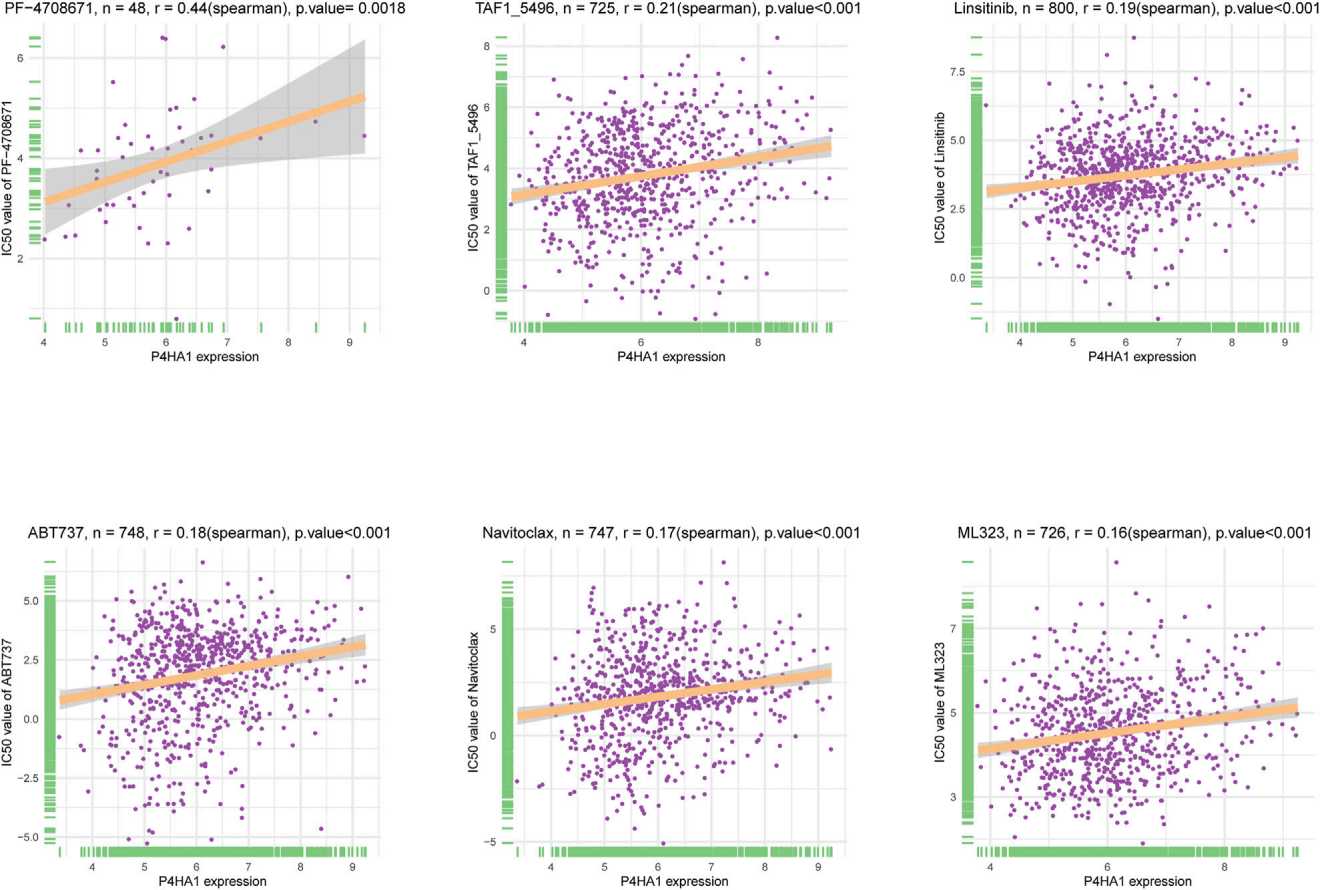
The correlation between P4HA1 expression and IC50 values of anti-cancer drugs. The correlation between P4HA1 expression and IC50 values of indicated anti-cancer drugs.

Additionally, we performed a correlation analysis between P4HA1 and IC50 values of 192 anticancer drugs. We found that patients with high P4HA1 expression might be resistant to most anticancer drugs, such as PF-4708671, TAF1_5496, Linsitinib, ABT737, Navitoclax, and ML323 (Figure 13). Supplementary Table S1 contains data on the correlation of IC50 value for various drugs.

In conclusion, our findings revealed that P4HA1 acts as an oncogene and a prognostic marker in pan-cancer and LUAD, and high P4HA1 expression might contribute to relative immunosuppressive microenvironment.

## Discussion

Tumor development and metastasis were found to be inextricably linked to the structure and function of tumor microenvironment (TME) (Yang et al., 2020). As the main component of tumor microenvironment, extracellular matrix (ECM) was considered intimately associated with tumor progression and immunotherapy resistance (Kesh et al., 2020). At present, cancer therapy focuses on developing new therapeutic strategies and discovering new drugs that target extracellular matrix. Collagens are major elements of ECM, and abnormally high deposition of collagens could remodel extracellular matrix. In addition, collagen could promote invasion and migration of malignant tumors (Maller et al., 2021). P4HA1 is the key enzyme in collagen. As a result, this study investigated the role of P4HA1 in pan-cancer and its impact on tumor microenvironment using bioinformatics analysis.

P4HA1 expression is elevated in many tumor types. Our study assessed P4HA1 expression and discovered elevated P4HA1 expression in 26 of 33 cancer types. Decreased P4HA1 expression was only observed in KICH and LAML (Leukemia). As illustrated in Figure 1, P4HA1 expression was the highest in bone marrow of normal tissues but reduced in LAML. Solid tumors had lower expression in normal tissues and increased expression in tumors, as observed in the case of kidney vs. KIRC, pancreas vs. PAAD, brain vs. GBM, and colon vs. COAD. The average expression value of P4HA1 was 3.62 in all normal tissues of GTEX. The expression value of P4HA1 was 7.29 in normal bone tissue, which was the highest in normal tissues. Pancreas was the normal tissue with the lowest average expression value (2.53). The average expression value of P4HA1 was 4.77 in all TCGA tumor tissues. The top 10 tumors with the highest average expression value were KIRC (5.98), UCS (5.70), LUSC (5.66), MESO (5.64), LUAD (5.43), BRCA (5.14), SARC (5.11), UCEC (5.11), SKCM (5.01), and GBM (4.99). KICH tumor had the lowest average expression value (3.19). All P4HA1 expression values in TCGA and GTEX are displayed in Supplementary Table S2. Comparing tumors and adjacent normal tissues in TCGA cohort, we also observed that P4HA1 was overexpressed in 12 cancers (Figures 2A–L). We further explored the relationship between P4HA1 and clinicopathological stages. The results revealed that elevated P4HA1 expression was associated with worse tumor stages in seven tumor types (Figures 2M–S). These findings indicate that these tumors also have a highly fibrotic phenotype and might be less responsive to immunotherapy.

Additionally, we discovered that mutation, methylation, and CNA of P4HA1 have important implications for P4HA1 mRNA expression. We found that melanoma patients had the highest alteration frequency of P4HA1 (>4%). Additionally, we revealed that alteration frequencies were 4, 3, 2.1, and 1.4% in esophagogastric, pancreatic cancer, invasive breast carcinoma, and lung adenocarcinoma, respectively. We further performed a correlation analysis between P4HA1 mutation and clinicopathological features of these cancers using cBiopotal database (http://www.cbioportal.org/). We discovered that P4HA1 mutation was associated with the pathological type and mutation count of invasive breast cancer. We also found that high CNA was one of the main causes of high P4HA1 expression in pan-cancer. Intriguingly, we observed that GBM was the only tumor in which DNA methylation status on P4HA1 promoter was positively linked to its mRNA expression (Figure 3C). Therefore, we queried the mexpress database (https://mexpress.be/) (Koch et al., 2015), a professional website for studying DNA methylation and expression data. P4HA1 expression in GBM was found to be positively correlated with methylation of multiple sites. The specific mechanism must be further investigated. To determine the prognostic value of P4HA1, we conducted UniCox and Kaplan-Meier survival analyses in TCGA cohort. Kaplan-Meier OS analysis revealed that increased P4HA1 expression indicated worse survival of patients with 18 cancer types (Figure 4). UniCox results indicated that P4HA1 acted as a risk factor for OS in patients with 12 cancer types. Based on hazard ratio, P4HA1 overexpression increased death risk in patients with different tumor types in the following order: KICH > THCA > UVM > MESO > KIRP > CESC > ACC > PAAD > LUAD > HNSC > SARC > BRCA.

GSEA results suggested that P4HA1 was mainly involved in cell cycle and immune-related pathways in pan-cancer. Numerous studies have demonstrated that high collagen deposition could impact or act as a barrier to tumor immune infiltration. Therefore, we used different immune cell infiltration data to analyze the correlation between P4HA1 expression and immune cell infiltration in pan-cancer. We found that infiltration levels of immunosuppressive cells, such as TAMs, nTregs, and iTregs, were positively correlated with P4HA1 expression in pan-cancer. In contrast, immune killer cells, such as activated NK cells and CD8 T cells, were negatively associated with P4HA1 expression based on three different analytical methods. At present, several studies have been reported on the role of P4HA1 overexpression in LUAD. Zhou et al. reported that P4HA1 overexpression was linked to poor survival and immune infiltrates in LUAD (Zhou et al., 2020). Robinson AD et al. revealed that P4HA1 was required for lung cancer cell growth and invasion, suggesting its potential as a valid therapeutic target in lung adenocarcinoma (Robinson et al., 2021). Additionally, our study focused on P4HA1 impact on lung adenocarcinoma. We found that P4HA1 was significantly overexpressed in lung adenocarcinoma compared with adjacent normal tissues and was associated with clinicopathological stages, poor OS, and DSS. These findings revealed that P4HA1, as an oncogene, was involved in lung adenocarcinoma progression, consistent with the results of Zhou and Robinson. Additionally, we discovered that high CNA was associated with high expression of P4HA1 in lung adenocarcinoma. In addition, we divided LUAD patients into two groups according to their median expression of P4HA1 and compared the differences of gene mutations between the two groups. The results indicated that patients with high P4HA1 expression had higher gene mutation frequency than those with low P4HA1 expression in LUAD (Figure 11). Besides, we found that patients with high P4HA1 expression had a higher tumor mutation burden (TMB). These findings indicated a potential association with immunotherapy efficacy. In addition, we analyzed the connection between P4HA1 expression and immune cells in lung adenocarcinoma using three distinct immune cell infiltration datasets and three distinct analysis methods. We found that P4HA1 expression was positively correlated with infiltration levels of immunosuppressive cells, such as TAMs, nTregs, and iTregs, but negatively linked to immune killer cells, such as activated NK cells and CD8 T cells, which were consistent with the results of Zhou et al., in 2020. More importantly, we found a similar trend in different tumors, such as STAD, COAD, KIRC, PRAD, CESC, etc. These findings implied that high P4HA1 expression was associated with tumor immune microenvironment inhibition in pan-cancer. We also proved that P4HA1 expression was positively correlated with TMB and MSI in these tumor types, implying a potential association with immunotherapy efficacy. Li M et al. reported that high P4HA1 levels could be employed as an early diagnostic and prognostic biomarker in patients with lung cancer, breast cancer, as well as head and neck cancer (Li et al., 2020b). Hu WM et al. identified that P4HA1 was a prognostic biomarker for high-grade gliomas (Hu et al., 2017). In addition, P4HA1 is supposed to relate closely with hypoxia and angiogenesis including HIF1a. Cao et al.revealed that the P4HA1-HIF1 a loop acted as a important regulator in glycolysis and oncogenesis and might serve as a potential therapy target for pancreatic cancer (Cao et al., 2019). Together with our study, all these studies demonstrated that P4HA1 was a potential biomarker for diagnosis and prognosis in pan-cancer.

Numerous studies have demonstrated that high collagen deposition mediated by P4HA1 expression could impact or act as a barrier to tumor immune infiltration. Larsen AMH et al. revealed that a high collagen density could instruct macrophages to acquire an immunosuppressive phenotype. This mechanism could reduce immunotherapy efficacy and explain the link between high collagen density and poor prognosis (Larsen et al., 2020). Kuczek DE et al. identified that high-density extracellular matrix down-regulated cytotoxic active markers while also up-regulating regulatory T cell markers. These changes involved autocrine TGF-β signal, accompanied by impaired ability of tumor-infiltrating T cells to kill autologous cancer cells. This new immune-modulatory mechanism could be essential for T cell activity suppression in tumor microenvironment (Kuczek et al., 2019). From GSEA results, we observed that elevated P4HA1 expression was positively linked to up-regulation of cell cycle signaling pathway in various tumors. This might be related to high collagen deposition mediated by high P4HA1expression. Collagen derived from ECM was known to regulate multiple signaling pathways that controlled cancer cell behavior by regulating mortality and invasiveness of cells (He et al., 2016). Recent studies have demonstrated that tumor cell surface DDR also played a crucial role in collagen-induced signaling pathway in tumor cells. Collagen DDR1 interaction induced phosphorylation and kinase activation of DDR1, which activated multiple downstream signaling pathways. Collagen DDR1-mediated Src kinase activation regulated proliferation and cell migration (Dejmek et al., 2003). DDR1 also activated proline-rich tyrosine kinase 2 (Pyk2) that ultimately induced N cadherin expression and regulated epithelial to mesenchymal transition (EMT) of cancer cells (Shintani et al., 2008). Improved understanding of collagen-mediated signaling pathways enables the development of new targets for cancer therapy.

Additionally, we conducted a correlation analysis between P4HA1 expression and IC50 of 192 anticancer drugs and discovered that patients with high P4HA1 expression might be resistant to most anticancer drugs, such as PF-4708671, TAF1_5496, linsitinib, ABT737, navitoclax, and ML323. Due to P4HA1 role in mediating high collagen deposition in tumor microenvironment and progression, new therapeutic strategies or small molecule inhibitors are under development to target collagen synthesis for cancer therapy. Discoidin domain receptor (DDR) was a collagen-activated receptor tyrosine kinase and has become an attractive target for anticancer therapy given its involvement in tumor growth, metastasis development, and tumor dormancy (Mehta et al., 2021). Whitney R et al. reported that a novel small molecule inhibitor (WRG-28) could inhibit tumor invasion and migration and metastatic breast tumor cell colonization in the lungs by targeting DDR2 (Grither and Longmore, 2018 Ru Dong et al. demonstrated that compound 6c was a potential DDR1 inhibitor deserving further investigation for cancer treatment (Dong et al., 2021).

In this paper, we conducted a comprehensive bioinformatics analysis of P4HA1 function in pan-cancer and identified P4HA1 as a potential prognostic biomarker and therapeutic target of pan-cancer. Because P4HA1-mediated high collagen deposition is critical in tumor microenvironment and progression, new therapeutic strategies or small molecule inhibitors are under development to target collagen synthesis for cancer therapy, which will be an important direction of cancer research in the future.

## Data Availability

The datasets presented in this study can be found in online repositories. The names of the repository/repositories and accession number(s) can be found in the article/Supplementary Material.

## Supplementary Material

Supplementary Table 1 P4HA1 expression and IC50 values of anti-cancer drugs.

Supplementary Table 2 P4HA1 expression values in TCGA and GTEx.

Supplementary Table 3 33 TCGA tumor abbreviation list and references

Supplementary Table 4 24 immune cell type abbreviation list

## Glossary

ACC: Adrenocortical carcinoma
BLCA: Bladder Urothelial Carcinoma
BRCA: Breast invasive carcinoma
CESC: Cervical squamous cell carcinoma and endocervical adenocarcinoma
CHOL: Cholangiocarcinoma
COAD: Colon adenocarcinoma
DLBC: Lymphoid Neoplasm Diffuse Large B-cell Lymphoma
ESCA: Esophageal carcinoma
GBM: Glioblastoma multiforme
HNSC: Head and Neck squamous cell carcinoma
KICH: Kidney Chromophobe
KIRC: Kidney renal clear cell carcinoma
KIRP: Kidney renal papillary cell carcinoma
LAML: Acute Myeloid Leukemia
LGG: Lower Grade Glioma
LIHC: Liver hepatocellular carcinoma
LUAD: Lung adenocarcinoma
LUSC: Lung squamous cell carcinoma
MESO: Mesothelioma
OV: Ovarian serous cystadenocarcinoma
PAAD: Pancreatic adenocarcinoma
PCPG: Pheochromocytoma and Paraganglioma
PRAD: Prostate adenocarcinoma
READ: Rectum adenocarcinoma
SARC: Sarcoma
SKCM: Skin Cutaneous Melanoma;
STAD: Stomach adenocarcinoma;
TGCT: Testicular Germ Cell Tumor;
THCA: Thyroid carcinoma
THYM: Thymoma
UCEC: Uterine Corpus Endometrial Carcinoma
UCS: Uterine Carcinosarcoma
UVM: Uveal Melanoma.
