# Supplementary material for: P4HA1, a Prognostic Biomarker that Correlates With Immune Infiltrates in Lung Adenocarcinoma and Pan-Cancer
Source: Front Cell Dev Biol. 2021 Dec 13;9:754580. doi: 10.3389/fcell.2021.754580 (PMC8710955; doi:10.3389/fcell.2021.754580)
Supplement: Supplementary file 2 [file Table2.DOCX]

| **English abbreviations** | English name |
| --- | --- |
| ACC | Adrenocortical carcinoma |
| BLCA | Bladder Urothelial Carcinoma |
| BRCA | Breast invasive carcinoma |
| CESC | Cervical squamous cell carcinoma and endocervical adenocarcinoma |
| CHOL | Cholangiocarcinoma |
| COAD | Colon adenocarcinoma |
| READ | Rectum adenocarcinoma |
| DLBC | Lymphoid Neoplasm Diffuse Large B-cell Lymphoma |
| ESCA | Esophageal carcinoma |
| GBM | Glioblastoma multiforme |
| LGG | Low Grade Glioma |
| HNSC | Head and Neck squamous cell carcinoma |
| KICH | Kidney Chromophobe |
| KIRC | Kidney renal clear cell carcinoma |
| KIRP | Kidney renal papillary cell carcinoma |
| LAML | Acute Myeloid Leukemia |
| LIHC | Liver hepatocellular carcinoma |
| LUAD | Lung adenocarcinoma |
| LUSC | Lung squamous cell carcinoma |
| MESO | Mesothelioma |
| OV | Ovarian serous cystadenocarcinoma |
| PAAD | Pancreatic adenocarcinoma |
| PCPG | Pheochromocytoma and Paraganglioma |
| PRAD | Prostate adenocarcinoma |
| SARC | Sarcoma |
| SKCM | Skin Cutaneous Melanoma |
| STAD | Stomach adenocarcinoma |
| TGCT | Testicular Germ Cell Tumors |
| THCA | Thyroid carcinoma |
| THYM | Thymoma |
| UCEC | Uterine Corpus Endometrial Carcinoma |
| UCS | Uterine Carcinosarcoma |
| UVM | Uveal Melanoma |

1.Abbreviation list

2. References on the role of P4HA1 in pan-cancer

| Tumor | References on the role of P4HA1 |
| --- | --- |
| BLCA | [Wa Hoong Chang](https://pubmed.ncbi.nlm.nih.gov/?term=Chang+WH&cauthor_id=31036064), [Donall Forde](https://pubmed.ncbi.nlm.nih.gov/?term=Forde+D&cauthor_id=31036064), [Alvina G Lai](https://pubmed.ncbi.nlm.nih.gov/?term=Lai+AG&cauthor_id=31036064).Dual prognostic role of 2-oxoglutarate-dependent oxygenases in ten cancer types: implications for cell cycle regulation and cell adhesion maintenance. Cancer Commun (Lond).. 2019 Apr 29;39(1):23 |
| COAD | Chen Z, Chen M, Xue Z, Zhu X. [Comprehensive Analysis of Gene Expression Profiles Identifies a P4HA1-Related Gene Panel as a Prognostic Model in Colorectal Cancer Patients.](https://pubmed.ncbi.nlm.nih.gov/34520234/) Cancer Biother Radiopharm. 2021 Sep 13 |
| GBM | Wang Q, Zhang J, Fang S, Wang J, Han X, Liu F, Jin G. [P4HA1 Down-Regulation Inhibits Glioma Invasiveness by Promoting M1 Microglia Polarization.](https://pubmed.ncbi.nlm.nih.gov/33727827/) Onco Targets Ther  . 2021 Mar 8;14:1771-1782  Zhu X, Liu S, Yang X, Wang W, Shao W, Ji T. P4HA1 as an unfavorable prognostic marker promotes cell migration and invasion of glioblastoma via inducing EMT process under hypoxia microenvironment. Am J Cancer Res. 2021 Feb 1;11(2):590-617.  Hu WM, Zhang J, Sun SX, Xi SY, Chen ZJ, Jiang XB, Lin FH, Chen ZH, Chen YS, Wang J, Yang QY, Guo CC, Mou YG, Chen ZP, Zeng J, Sai K. Identification of P4HA1 as a prognostic biomarker for high-grade gliomas. Pathol Res Pract. 2017 Nov;213(11):1365-136 |
| HNSC | [Mingjie Li](https://pubmed.ncbi.nlm.nih.gov/?term=Li+M&cauthor_id=32150689), [Fudan Wu](https://pubmed.ncbi.nlm.nih.gov/?term=Wu+F&cauthor_id=32150689), [Qinqin Zheng](https://pubmed.ncbi.nlm.nih.gov/?term=Zheng+Q&cauthor_id=32150689), [Yinlong Wu](https://pubmed.ncbi.nlm.nih.gov/?term=Wu+Y&cauthor_id=32150689), [Yan'an Wu](https://pubmed.ncbi.nlm.nih.gov/?term=Wu+Y&cauthor_id=32150689). Identification of Potential Diagnostic and Prognostic Values of P4HA1 Expression in Lung Cancer, Breast Cancer, and Head and Neck Cancer. DNA Cell Biol. 2020 May;39(5):909-917.  [Baoling iu](https://pubmed.ncbi.nlm.nih.gov/?term=Liu+B&cauthor_id=34221975), [Quanping Su](https://pubmed.ncbi.nlm.nih.gov/?term=Su+Q&cauthor_id=34221975), [Jianhua Ma](https://pubmed.ncbi.nlm.nih.gov/?term=Ma+J&cauthor_id=34221975), [Cheng Chen](https://pubmed.ncbi.nlm.nih.gov/?term=Chen+C&cauthor_id=34221975), [Lijuan Wang](https://pubmed.ncbi.nlm.nih.gov/?term=Wang+L&cauthor_id=34221975), [Fengyuan Che](https://pubmed.ncbi.nlm.nih.gov/?term=Che+F&cauthor_id=34221975), [Xueyuan Heng](https://pubmed.ncbi.nlm.nih.gov/?term=Heng+X&cauthor_id=34221975). Prognostic Value of Eight-Gene Signature in Head and Neck Squamous Carcinoma. Front Oncol. 2021 Jun 18;11:657002. |
| LIHC | [Guoxing Feng](https://pubmed.ncbi.nlm.nih.gov/?term=Feng+G&cauthor_id=26966067), [Hui Shi](https://pubmed.ncbi.nlm.nih.gov/?term=Shi+H&cauthor_id=26966067), [Jiong Li](https://pubmed.ncbi.nlm.nih.gov/?term=Li+J&cauthor_id=26966067), [Zhe Yang](https://pubmed.ncbi.nlm.nih.gov/?term=Yang+Z&cauthor_id=26966067), [Runping Fang](https://pubmed.ncbi.nlm.nih.gov/?term=Fang+R&cauthor_id=26966067), [Lihong Ye](https://pubmed.ncbi.nlm.nih.gov/?term=Ye+L&cauthor_id=26966067), [Weiying Zhang](https://pubmed.ncbi.nlm.nih.gov/?term=Zhang+W&cauthor_id=26966067), [Xiaodong Zhang](https://pubmed.ncbi.nlm.nih.gov/?term=Zhang+X&cauthor_id=26966067). MiR-30e suppresses proliferation of hepatoma cells via targeting prolyl 4-hydroxylase subunit alpha-1 (P4HA1) Mrna. Biochem Biophys Res Commun. 2016 Apr 8;472(3):516-22. |
| LUAD | [Haiting Zhou](https://pubmed.ncbi.nlm.nih.gov/?term=Zhou+H&cauthor_id=33299876), [Yi He](https://pubmed.ncbi.nlm.nih.gov/?term=He+Y&cauthor_id=33299876), [Lingling Li](https://pubmed.ncbi.nlm.nih.gov/?term=Li+L&cauthor_id=33299876), [Cheng Wu](https://pubmed.ncbi.nlm.nih.gov/?term=Wu+C&cauthor_id=33299876), [Guoqing Hu](https://pubmed.ncbi.nlm.nih.gov/?term=Hu+G&cauthor_id=33299876). Overexpression of P4HA1 Is Correlated with Poor Survival and Immune Infiltrates in Lung Adenocarcinoma. Biomed Res Int. 2020 Nov 24;2020:8024138  Robinson AD, Chakravarthi BVSK, Agarwal S, Chandrashekar DS, Davenport ML, Chen G, Manne U, Beer DG, Edmonds MD, Varambally S. [Collagen modifying enzyme P4HA1 is overexpressed and plays a role in lung adenocarcinoma.](https://pubmed.ncbi.nlm.nih.gov/34049151/) Transl Oncol. 2021 Aug;14(8):101128.  [Wai Hoong Chang](https://pubmed.ncbi.nlm.nih.gov/?term=Chang+WH&cauthor_id=31036064), [Donall Forde](https://pubmed.ncbi.nlm.nih.gov/?term=Forde+D&cauthor_id=31036064), [Alvina G Lai](https://pubmed.ncbi.nlm.nih.gov/?term=Lai+AG&cauthor_id=31036064). Dual prognostic role of 2-oxoglutarate-dependent oxygenases in ten cancer types: implications for cell cycle regulation and cell adhesion maintenance. Cancer Commun (Lond). 2019 Apr 29;39(1):23 |
| PAAD | Cao XP, Cao Y, Li WJ, Zhang HH, Zhu ZM. P4HA1/HIF1α feedback loop drives the glycolytic and malignant phenotypes of pancreatic cancer.Biochem Biophys Res Commun. 2019 Aug 27;516(3):606-612. |
